# Determination of major sialylated N-glycans and identification of branched sialylated N-glycans that dynamically change their content during development in the mouse cerebral cortex

**DOI:** 10.1007/s10719-014-9566-2

**Published:** 2014-11-23

**Authors:** Tomohiro Torii, Takeshi Yoshimura, Mai Narumi, Seiji Hitoshi, Yoshie Takaki, Shuichi Tsuji, Kazuhiro Ikenaka

**Affiliations:** 1 Department of Physiological Sciences, School of Life Sciences, The Graduate University for Advanced Studies (SOKENDAI), Shonan Village, Hayama, Kanagawa 240-0193 Japan; 2 Division of Neurobiology and Bioinformatics, National Institute for Physiological Sciences, National Institutes of Natural Sciences, 5-1 Higashiyama, Myodaiji-cho, Okazaki, Aichi 444-8787 Japan; 3 Institute of Glycoscience, Tokai University, 4-1-1 Kitakaname, Hiratsuka, Kanagawa 259-1292 Japan; 4 Present Address: Department of Pharmacology, National Research Institute for Child Health and Development, 2-10-1 Okura, Setagaya, Tokyo 157-8535 Japan; 5 Present Address: Department of Integrative Physiology, Shiga University of Medical Science, Seta Tsukinowa-cho, Otsu, 520-2192 Japan

**Keywords:** Sialylated N-glycan, Mouse brain development, Pyridylamination, Siglec

## Abstract

Oligosaccharides of glycoproteins expressed on the cell surface play important roles in cell-cell interactions, particularly sialylated N-glycans having a negative charge, which interact with sialic acid-binding immunoglobulin-like lectins (siglecs). The entire structure of sialylated N-glycans expressed in the mouse brain, particularly the linkage type of sialic acid residues attached to the backbone N-glycans, has not yet been elucidated. An improved method to analyze pyridylaminated sugar chains using high performance liquid chromatography (HPLC) was developed to determine the entire structure of sialylated N-linked sugar chains expressed in the adult and developing mouse cerebral cortices. Three classes of sialylated sugar chains were prevalent: 1) N-glycans containing α(2-3)-sialyl linkages on a type 2 antennary (Galβ(1-4)GlcNAc), 2) sialylated N-glycans with α(2-6)-sialyl linkages on a type 2 antennary, and 3) a branched sialylated N-glycan with a [Galβ(1-3){NeuAcα(2-6)}GlcNAc-] structure, which was absent at embryonic day 12 but then increased during development. This branched type sialylated N-glycan structure comprised approximately 2 % of the total N-glycans in the adult brain. Some N-glycans (containing type 2 antennary) were found to change their type of sialic acid linkage from α(2-6)-Gal to α(2-3)-Gal. Thus, the linkages and expression levels of sialylated N-glycans change dramatically during brain development.

**Electronic supplementary material:**

The online version of this article (doi:10.1007/s10719-014-9566-2) contains supplementary material, which is available to authorized users.

## Introduction

Sugar chains envelop the vast majority of the cell surface and are considered to play significant roles in cell-cell and extracellular matrix interactions as mediators and as signal transducers. Increasing evidence suggests that glycosylation is critical to various biological functions and requires the coordinated action of glycosyltransferases [1, 2]. Others and we have studied developmental changes in the gene expression of sugar chain-metabolizing enzymes and the structure of glycoconjugates and found that the expression of glycoconjugates including N-glycans and glycosphingolipids changes dramatically during development [3, 4], differentiation [5], and oncogenic transformation [6]. However, so far we have only been able to analyze the structure of N-linked sugar chains after removal of sialic acid residues due to the complexity of glycosylation analysis.

Sialic acids are widely distributed as terminal sugars that coat the cell surface and participate in various biological processes, including cell-cell communications, cell migration, adhesion, inflammation, immune defense, and cancer metastasis [7]. In the rodent brain, 40 % of total N-glycan is sialylated and approximately 10 % of these N-glycans carry polysialic acids (PSA), which are partly comprised of PSA on neural cell adhesion molecules (N-CAM) [8]. Growing evidence suggests that structures containing sialic acid play particularly important roles in cell-cell and extracellular matrix interactions [9–12]. Proteins that recognize and bind to a certain structure of sugar chains are called lectins. Among the lectins, those that bind to sialic acid-containing structures are named “siglecs” (sialic acid-binding immunoglobulin-like lectins), demonstrating the significance of this type of lectin. Siglecs can recognize not only the sialic acid residue but also the sugar residue next to it [13, 14]. They also distinguish the linkage between sialic acid and the next sugar residue [15]. Thus, siglecs that bind to α(2-3)-sialic acid are distinct from those that bind to α(2-6)-sialic acid. The α(2-3)-sialic acid binding proteins such as CHL1, NrCAM, and NgCAM, belong to the L1 super-family and have all been shown to be neurite outgrowth promoters and/or mediators of neuron–glia interactions [16]. Myelin-associated glycoprotein (MAG), a member of the siglecs recognizing α(2-3)-sialic acid, is involved not only in myelin formation, but also in myelin maintenance [17]. It has received particular attention because it enhances *in vitro* neurite outgrowth at early developmental stages but inhibits neurite outgrowth in adults [18]. MAG might thereby contribute to a non-conducive environment for regeneration in the adult mammalian nervous systems. Therefore, it is extremely important to determine the structure of sialic acid-containing sugar chains in the adult brain as well as changes in glycosylation during development to understand the significance of interactions between siglecs and sialic acid-containing sugar chains. Brain ganglioside structures have been well analyzed and their developmental profile of expression is known [19, 20]. However, structures of N-linked sugar chains during brain development remain poorly understood. Therefore, we aimed to identify sialylated N-glycans whose expression levels vary dramatically during brain development.

In an earlier report, the structure of sialylated N-glycans in the adult rat brain was thoroughly analyzed and quantified [8]. Sialylated N-glycans were found to carry predominantly α(2-3)-linked sialic acid in the adult rat brain as compared with α(2-6)-linked sialic acid [8, 21]. The structures of most of the sialic acid-containing N-linked sugar chains were also determined. However, the quantification of each sugar chain was performed using MALDI-TOF mass spectrometry. Quantification of different substances using this method is difficult since their ionization efficiencies can differ significantly, and furthermore the developmental profile of sugar chains was not investigated. Moreover, there was a critical lack of information regarding the linkage of sialic acid bound to each N-linked sugar chain, because permethylation analysis of the entire set of brain sugar chains was performed without purification prior to the linkage analysis.

We have been developing a systematic method to analyze N-glycan expression patterns present in whole tissues without glycoprotein purification after removal of sialic acid residues [22–25]. Here, we report a simple method to analyze sialic acid-containing N-glycans, and we systematically analyze expression levels and linkages of sialylated N-glycans in the developing mouse cerebral cortex using this system. This study identified one sialylated N-glycan having a branched sialic acid structural feature. This N-glycan contains a sialic acid attached to the *N*-acetylglucosamine residue of the Galβ(1-3)-GlcNAc- moiety at the non-reducing end *via* an α(2-6)-linkage, with no sialic acid attached to the galactose residue [Galβ(1-3){NeuAcα(2-6)}GlcNAc-], which we termed the 6-sialyl Lewis C structure. This branched sialylated structure changed its content dynamically during development in the mouse brain, suggesting that N-glycans with 6-sialyl Lewis C plays important roles during brain development.

## Materials and methods

### Materials and chemicals

Anhydrous hydrazine was purchased from Tokyo Chemical Industry (Tokyo, Japan), 2-aminopyridine was from Kanto Chemical (Tokyo, Japan), and dimethylamine borane was from Wako (Osaka, Japan). Graphite carbon columns (GL-Pak carbograph, Cat. No. 5010–23005) were purchased from GL Science (Tokyo, Japan). Cellulose cartridge columns were from Takara Bio (Cat. No. 4404; Otsu, Japan) and GL Science (Cat. No. 5010–11130). Microgranular cellulose for packed cellulose columns was from Sigma (St. Louis, MO, USA). Neuraminidase derived from *Arthrobacter ureafaciens* was purchased from Nacalai Tesque (Kyoto, Japan). α2,3-sialidase, specific for α(2-3)-NeuAc, was from New England BioLabs (Ipswich, MA, USA). Pyridylaminated (PA)-sugar chains used as a standard were purchased from Takara Bio and Seikagaku Corporation (Tokyo, Japan).

### Animals

ICR mice were purchased from Japan SLC (Hamamatsu, Japan). All experiments were carried out with permission of the institutional Animal Research Committee of the National Institute for Physiological Sciences.

### Purification and pyridylamination of sugar chains

N-glycan purification and pyridylamination were performed as described previously [23–25]. Briefly, ICR mice at various developmental stages (embryonic day 12 (E12), E16, postnatal day 0 (P0), P7) or adult (12 weeks (12w)) were sacrificed and their cerebral cortices were quickly removed and washed with ice-cold phosphate-buffered saline (PBS). Tissues were homogenized in a nine-fold volume of acetone using a polytron homogenizer. After acetone precipitation, samples were lyophilized before use. Each lyophilized sample (2 mg) was hydrazinolyzed (100 °C, 10 h). N-glycan purification and in-column N-acetylation was performed using graphite carbon columns. The reducing ends of the liberated glycans were tagged with the fluorophore 2-aminopyridine to aid detection *via* high-performance liquid chromatography (HPLC) analyses. Excess reagents were removed and PA-N-glycans were purified using cellulose columns. To analyze N-glycans at each stage, the samples were prepared from more than ten mice at each developmental stage and three adult mice for each experiment.

### N-glycan analysis and separation by HPLC

To separate neutral-, mono-, di-, tri-, and tetra-sialyl N-glycans, PA-N-glycans were passed through an anion-exchange column (Mono Q 5/50 GL, GE Healthcare, Little Chalfont, UK) using HPLC or a Microgranular DE52-packed column (Whatman, GE Healthcare). Anion-exchange column HPLC purification was performed at a flow rate of 1.0 ml/min at room temperature. The mobile phase consisted of solvent A (distilled water adjusted to pH 9.0 with aqueous ammonia) and solvent B (0.5 M ammonium acetate titrated to pH 9.0 with aqueous ammonia). Neutral N-glycans were collected in the non-adsorbed fraction. PA-sugar chains were detected at excitation and emission wavelengths of 310 nm and 380 nm, respectively (FP-2025 Plus, Jasco Corporation, Hachioji, Japan).

Sialylated PA-N-glycans were treated with neuraminidase at 37 °C for 14 h in 50 mM ammonium acetate pH 5.0 to cleave sialic acids, followed by heating at 100 °C for 5 min and filtering through a 0.20 μm spin filter (Ultrafree-MC LG, Millipore, Billerica, MA, USA).

Analyses of neutral PA-N-glycans using HPLC were performed as described previously [3, 23–26]. Briefly, PA-tagged N-glycans of varying sizes were separated by HPLC using a normal-phase (NP)-column (Shodex Asahipak NH2P-50 4E, 4.6 × 250 mm, Showa Denko K.K., Tokyo, Japan) at a flow rate of 0.6 ml/min at 30 °C. PA-N-glycans were detected at an excitation wavelength of 310 nm and an emission wavelength of 380 nm using a fluorescence detector. Each detected PA-N-glycan was further analyzed by reverse-phase (RP) HPLC. RP-HPLC was performed on a Develosil C30-UG-5 column (4.6 × 150 mm, Nomura Chemical, Seto, Japan) at a flow rate of 0.5 ml/min at 30 °C. PA-sugar chains were detected at excitation and emission wavelengths of 320 and 400 nm, respectively. N-glycan structures were identified by calculating the Mannose-Unit value from NP-HPLC, and the Glucose-Unit value from RP-HPLC, as described previously [26, 27], or by comparison with known standards and sequential exoglycosidase digestion.

RP-HPLC for purification of sialylated N-glycans was performed on a Develosil ODS-5 column (4.6 × 250 mm, Nomura Chemical) at a flow rate of 1.0 ml/min. The column was equilibrated with 100 mM acetic acid titrated to pH 4.0 with triethylamine containing 0.05 % 1-butanol. After injecting each sample, the concentration of 1-butanol was increased linearly to 0.4 % over 90 min. PA-sugar chains were detected at an excitation wavelength of 320 nm and an emission wavelength of 400 nm.

### Data quantification and analysis of PA-sugar chains

PA-N-glycans were quantified and analyzed as described previously [3, 23–26]. HPLC chromatogram data were analyzed using Unipoint software (Gilson Inc., Middleton, WI, USA), LC station software (Shimadzu, Kyoto, Japan) and Empower2 software (Waters, Milford, MA, USA).

### Matrix assisted laser desorption ionization time-of-flight mass spectrometry (MALDI/TOF-MS)

The molecular masses of PA-sugar chains and their isobaric monosaccharide compositions were determined by MALDI/TOF-MS. The PA-sugar chains were dissolved in water. One microliter of matrix solution (10 mg/ml 2,5-dihydroxybenzoic acid in 30 % acetonitrile) was applied on the target spot of plate, and 1 microliter of sample solution was added, and then dried by warm air. MALDI/TOF mass spectra of the samples were acquired using a REFREX spectrometer (Bruker-franzen, Germany) in the positive and reflector mode at an acceleration voltage of 20 kV and delayed ion extraction. Standard PA-sugar chains were used to achieve a two-point external calibration for mass assignment of ions. The mass spectra shown are the sum of at least 30 laser shots.

### Exoglycosidase digestion

Purified PA-N-glycans were digested for 3 h at 37 °C using the following each enzyme: *Xanthomonas manihotis* β1,3-galactosidase (New England BioLabs), specific for β(1-3)-Gal, in 50 mM sodium acetate pH 4.5, 100 μg/ml BSA; *Diplococcus pneumoniae* β-galactosidase (Roche Diagnostics, Basel, Switzerland), specific for β1,4-Gal, in 50 mM sodium acetate pH 6.0; α1,3/4-L-fucosidase (Takara Bio), specific for α1,3/4-Fuc, in 100 mM sodium phosphate buffer pH 6.0; bovine kidney α1,6-fucosidase (Prozyme, Hayward, CA, USA), specific for α1-6 > 1-2/3/4-Fuc, in 100 mM sodium phosphate buffer pH 6.0. Neuraminidase, specific for α2,3/6/8-NeuAc, was incubated with PA-N-glycans for 14 h at 37 °C in 50 mM ammonium acetate pH 5.0. α2,3-sialidase, specific for α(2-3)-NeuAc, was incubated with PA-N-glycans for 14 h at 37 °C in 50 mM sodium citrate pH 6.0, 100 mM NaCl, 100 μg/ml BSA. These samples were heated at 100 °C for 5 min. The reaction mixture was centrifuged at 2,300 × g for 10 min, followed by filtering through a 0.20 μm spin filter (Ultrafree-MC LG, Millipore).

### SNA lectin affinity chromatography

SNA agarose beads (Vector Laboratory, Burlingame, CA, USA) were packed into a 50 × 10 mm assist mini-column. One milliliter of the sample was applied to the SNA column after it had been equilibrated with buffer A, which consisted of 20 mM Tris–HCl (pH 7.5) containing 0.15 M NaCl, 1 mM MnCl_2_, 1 mM CaCl_2_. Unbound oligosaccharides were washed away with buffer A, and the bound sugar chains were then eluted from the column with 0.1 M lactose in buffer A.

## Results

### Analysis of sialylated N-glycans in mouse cortices

**Fig. 1:**
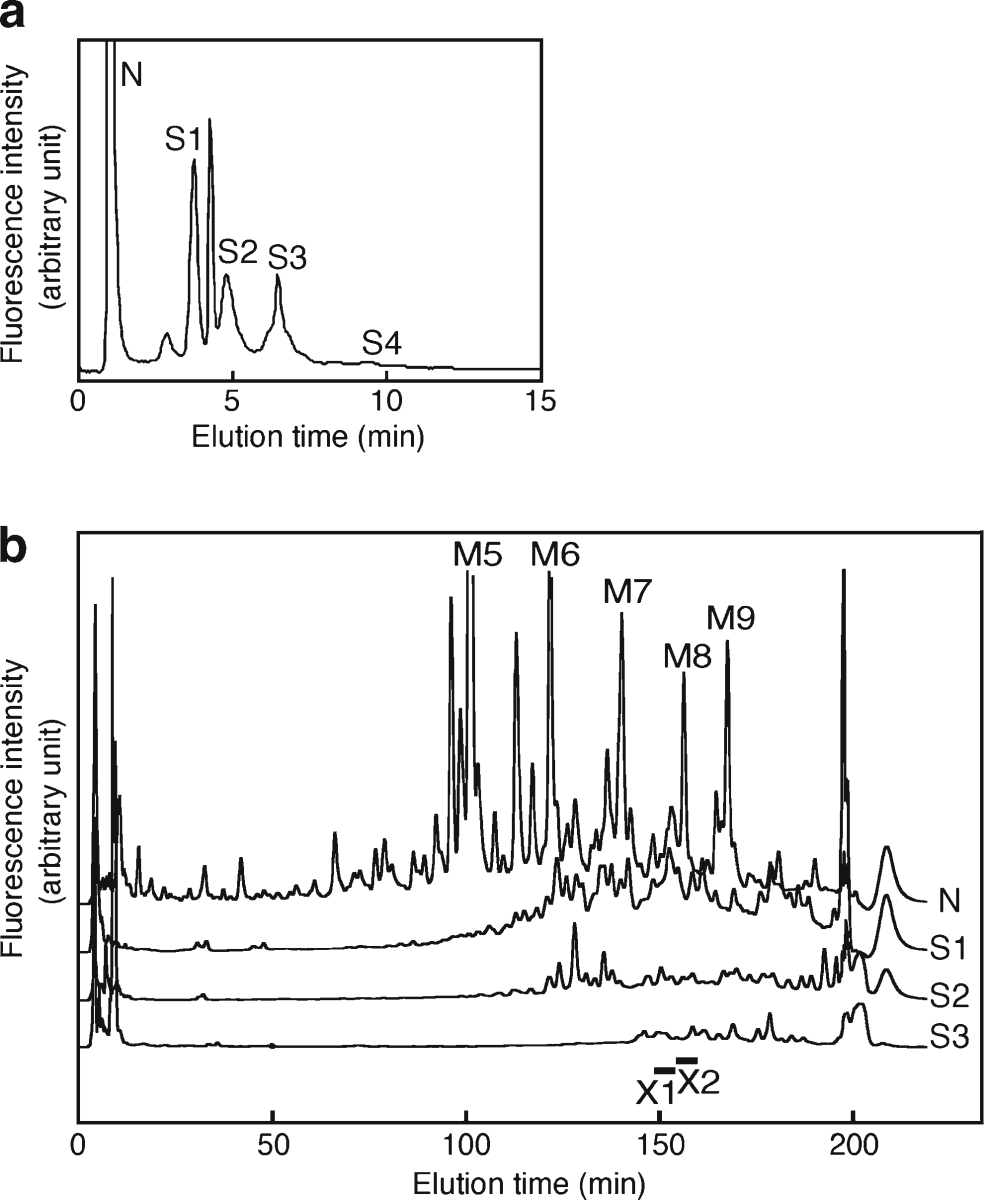
Simple approaches for structural analysis of sialylated N-glycans using HPLC. **a** Mono Q HPLC chromatogram from the P0 mouse cortex. N, S1, S2, S3 and S4 indicate the elution positions of neutral, monosialo, disialo, trisialo and tetrasialo PA-oligosaccharides, respectively. The fractions (N, S1, S2 and S3) were collected separately. **b** NP-chromatograms of neutral and desialylated PA-sugar chains from the P0 mouse cerebral cortex. After fractionation on a Mono Q column, N-glycans in the N fraction was directly analyzed by NP-HPLC. After N-glycans in the S1, S2 and S3 fractions were desialylated with neuraminidase, the samples were analyzed by NP-HPLC. M5-M9 indicate high-mannose type sugar chains. The bars with X1 and X2 indicate the elution positions of X1 and X2, respectively. Results are representative of more than six experiments

We have previously identified the structures of neutral and desialylated N-glycans that comprised more than 1 % of the total N-glycan content in the developing mouse brain [3]. The present study further identifies the structures of sialylated N-glycans that are abundantly present in the mouse cortices and the developmental profile of their content. P0 mouse brains were acetone precipitated and 2 mg each of dried precipitate was subjected to hydrazinolysis. They were further processed for purification and pyridylamination as described in Materials and Methods. PA-N-glycans from mouse brain were applied to a Mono Q column, which separates sugar chains according to their negative charge (Fig. 1a). Fractions corresponding to neutral N-glycan-containing fraction (N), mono-sialyl N-glycan-containing fraction (S1), di-sialyl N-glycan-containing fraction (S2) and so on were isolated (Fig. 1a). Amounts of N-glycans eluting from the S4 fraction or from fractions containing more sialic acids were very low compared to those from other fractions. There is a sharp peak corresponding to monosulfated N-glycans between S1 and S2. A low peak between N and S1 was derived from contaminants that were not removed during N-glycan purification. Over 50 % of the total N-glycans were confirmed to have a neutral charge. The N fraction was collected and then N-glycans in the N fraction were directly applied to NP-HPLC, which revealed that they were composed primarily of oligomannose-type sugar chains, which constitute 45.5 % of the total N-linked sugar chains in the adult mouse cerebral cortex [3]. These highly abundant high mannose-type sugar chains (M5 to M9) can be detected on NP-HPLC chromatograms (Fig. 1b).

**Fig. 2:**
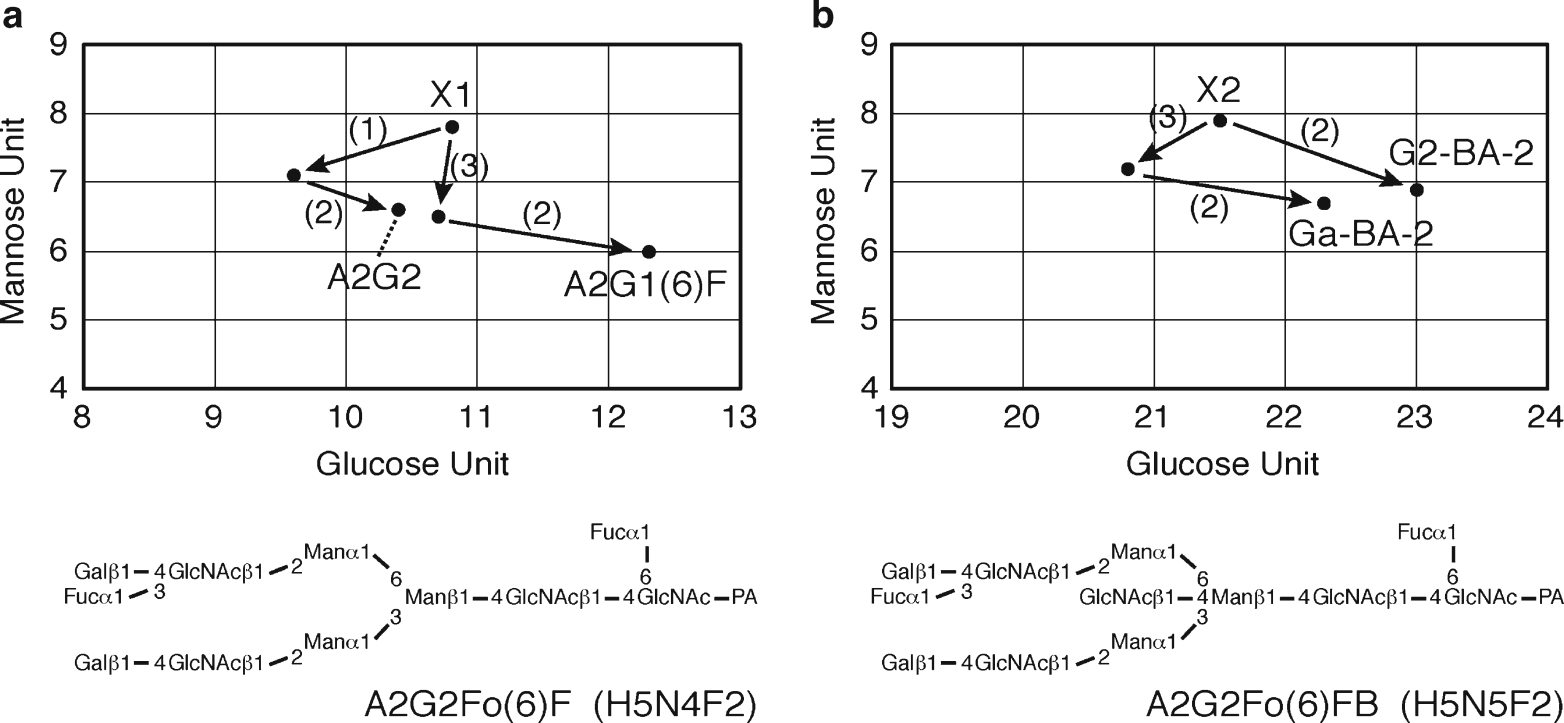
Structural determination of N-glycans by exoglycosidase digestions. Schemes showing 2D-mapping of desialylated N-glycans after exoglycosidase treatment. On this map, the *horizontal* and *vertical* axes correspond to GU (RP-HPLC) and MU (NP-HPLC), respectively. Trajectories for enzymatic digestions are indicated by *arrows* with *solid lines*. The numbers of the plotted points indicate the oligosaccharide structures. The nomenclature of the structures is shown in the footnote. Composition: H = hexose, N = GlcNAc, F = Fucose. Enzymes: α1,6-fucosidase (1), α1,3/4-L-fucosidase (2), and β1,4-galactosidase (3) . **a** Determination of X1 structure. **b** Determination of X2 structure

To characterize the sialylated N-glycans, the fractions (S1, S2 and S3) were collected separately. The samples were desialylated with neuraminidase and then applied to a DE52 column. The flow-through fraction was subjected to NP-HPLC; however, the peaks were still incompletely separated and could not be identified or quantified at this point (Fig. 1b). The eluate was therefore collected following the NP-HPLC into 30 fractions, which were subjected to RP-HPLC. We have improved the separation achieved by NP-HPLC [25] and the number of fractions was thereby increased from 10 to 30, improving the resolution of samples. Using this improved method, two new N-glycans present at a relatively high abundance (more than 0.1 % of total N-glycan content) were identified, which were not found in our previous analysis [3]; they have tentatively been named X1 and X2. The structures of these two sugar chains were determined as described below. A two-dimensional (2D) map of PA-N-linked sugar chains expressed in adult mouse brain was constructed using as indices the mannose units (MU) measured by NP-HPLC and the glucose units (GU) measured by RP-HPLC. The structures were identified by exoglycosidase digestion and MALDI/TOF/MS as follows. Firstly, the molecular masses of the PA-sugar chains were determined by MALDI/TOF-MS. The X1 composition estimated from the measured mass number was deoxyHex_2_Hex_5_HexNAc_4_-PA (Table 1). The PA-N-glycan X1 showed an elution time in MU of 7.8 on NP-HPLC and in GU of 10.8 on RP-HPLC. The sugar was susceptible to digestion with α1,6-fucosidase (Fig. 2a (1)). In this case, one fucose residue was released, and the product was mapped to the position (GU 9.6, MU 7.1) on the 2D-map. Further digestion was performed with α1,3/4-fucosidase (Fig. 2a (2)), resulting in the release of one fucose residue. This digested product was mapped to the position (GU 10.4, MU 6.6), which co-eluted on RP-HPLC with the standard PA-N-glycan, A2G2. The product of β1,4-galactosidase digestion (Fig. 2a (3)) was further digested with α1,3/4-fucosidase. This product (GU 12.3, MU 6.0) co-eluted with the standard, A2G1(6)F. Thus the structure of PA-N-glycan X1 was determined to be A2G2Fo(6)F (Fig. 2a, structures shown in Table 2).

**Table 1:** Mass analysis of X1 and X2. *Average mass calculated from the *m*/*z* values of [M + Na]^+^ ions for PA-oligosaccharides

N-glycan	Observed *m*/*z* (as a Na^+^ adduct)	Measured mass*	Monoisotopic mass	Estimated composition	Abbreviation
X1	2034.31	2011.32	2010.76	deoxyHex_2_Hex_5_HexNAc_4_-PA	A2G2Fo(6)F
X2	2237.45	2214.46	2213.84	deoxyHex_2_Hex_5_HexNAc_5_-PA	A2G2Fo(6)FB

**Table 2:**
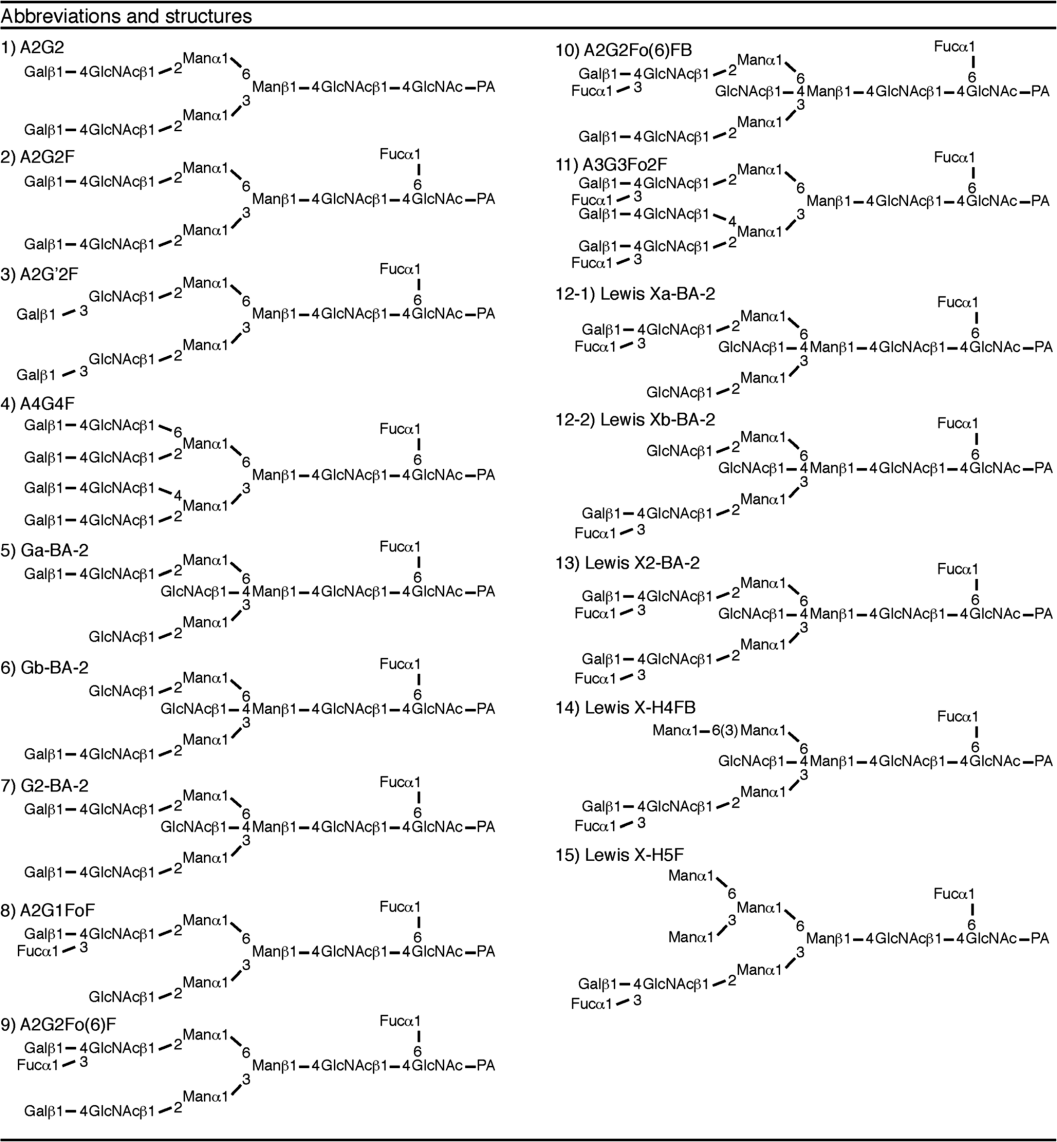
Structures of N-glycans and their abbreviations. All structures are shown as pyridylaminated (PA-) forms. The nomenclature of oligosaccharide structures was described in Footnotes and reported previously [3, 26]

The X2 composition estimated from the measured mass number was deoxyHex_2_Hex_5_HexNAc_5_-PA (Table 1). The PA-N-glycan X2 (GU 21.5, MU 7.9) was digested with β1,4-galactosidase (Fig. 2b (3)). In this case, one galactose residue was released, and the product was mapped to the position (GU 20.8, MU 7.2) on the 2D-map. Further digestion was performed with α1,3/4-fucosidase (Fig. 2b (2)), resulting in the release of one fucose residue. This digested product was mapped to the position (GU 22.3, MU 6.7), which co-eluted on RP-HPLC with the standard PA-N-glycan, Ga-BA-2. X2 was digested with α1,3/4-fucosidase. This product (GU 23.0, MU 6.9) co-eluted with the standard, G2-BA-2. Thus, the structure of the PA-sugar chain X2 was determined to be A2G2Fo(6)FB (Fig. 2b).

**Fig. 3:**
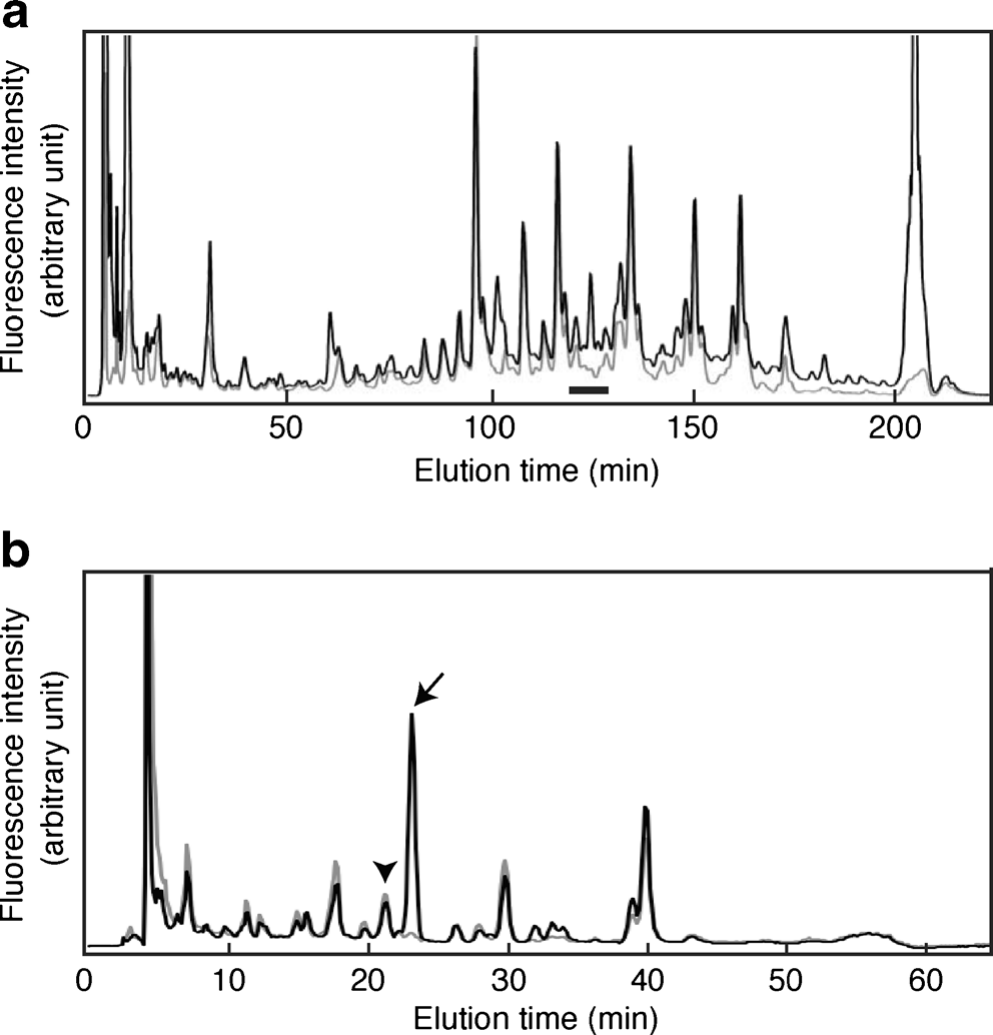
Practical approaches for quantitative analysis of sialylated N-glycans using HPLC. Neutral and asialo N-glycans, which were treated with neuraminidase, were separated and fractionated by Mono Q HPLC. Fractions were then analyzed by NP-HPLC **a** and a fraction indicated by the bar was collected. This fraction was separated by RP-HPLC for further analysis **b**. The *black line* indicates the chromatogram of neutral and desialylated PA-oligosaccharides derived from the P0 mouse brain and the *gray line* indicates that of neutral sugar chains. From the composite image of two chromatograms, sialylated sugar chains can be calculated as the difference in the peak areas. *Arrow* indicates the elution position of A2G2, *arrowhead* indicates the elution position of A1G1FoFB. Results are representative of more than six experiments

All other peaks whose content was over 0.1 % of the total N-glycan content had been identified previously [3, 8]. Thus it became possible to quantify major N-glycans contained in each Mono Q fraction (N, S1 to S4) and to determine the sialic acid linkages of each N-glycan. However, since several chromatographic separation steps were employed to separate the sugar chains, we could not reliably compare the levels of N-glycans contained in different Mono Q fractions. Thus we developed a more practical approach for quantitative analysis of sialylated N-glycans. The PA-sugar chain mixture was treated with neuraminidase and then applied to Mono Q HPLC, and the flow-through fraction was collected. This fraction contains neutral sugar chains and desialylated sugar chains (N + D fraction). Fig. 3a shows a differential NP-HPLC pattern obtained from the N + D (solid line) and N (grey line) fractions of the P0 mouse brain. The amount of sialylated sugar chains can be estimated by comparing the areas under each peak obtained from the N fraction with that from the N + D fraction. However, the separation thus achieved was insufficient, so these fractions were then subjected to RP-HPLC. Figure 3b shows an RP-HPLC chromatogram of a fraction indicated by a bar in Fig. 3a; the sialylated component of each sugar chain is now visible. For example, A2G2 (indicated by an arrow) was detected only in the N + D fraction, but not in the N fraction. This indicates that A2G2 was completely sialylated. On the other hand, a sugar chain indicated by an arrowhead is equally abundant in the N and N + D fractions, indicating that this sugar chain is not sialylated at all.

We examined the sialylated proportions of highly abundant N-glycans in mouse brains (structures shown in Table 2). To analyze each N-glycan quantitatively, N-glycans were separated by a three-dimensional HPLC system using Mono Q, NP-, and RP-columns. Since high-mannose type N-glycans are not sialylated [16, 28], highly abundant complex type and hybrid type N-glycans were investigated [3]. Most sialylated N-glycans present in the developing and adult mouse cortices were systematically separated and quantified using the system (Table 3). The sialylated proportions of A2G2 at E12, A2G’2F at E12, A2G2Fo(6)FB at E12 and A3G3Fo2F at E12, E16, P0 and P7 were not examined because the expression of these N-glycans was not detected, as previously reported [3]. The N-glycan content in the N + D fraction was subtracted by its content in the N fraction and was termed the C^α2,3/6/8^ value, which represents the neuraminidase sensitive portion of a given N-glycan. Most N-glycans containing outer fucose residues (Fo), namely A2G1FoF, Lewis X(a + b)-BA-2, Lewis X2-BA-2, Lewis X-H4FB, and Lewis X-H5F, had a C^α2,3/6/8^ value of 0, meaning that they did not contain sialic acid residues (Table 3). These results indicate that the Lewis X epitope present in the mouse cortex from any developmental stage is not sialylated. About 70 % of A2G2Fo(6)F and A2G2Fo(6)FB were sialylated (Table 3); these contain one Lewis X epitope and one galactose residue at their non-reducing ends. We speculate that the Gal-containing antennary of these N-glycans was sialylated. Therefore, it is likely that developing and adult mouse cortices contain no sialyl Lewis X moiety.

**Table 3:** Proportion of sialic acid-containing N-glycans in developing and adult cortices. Quantification of the neuraminidase or α2,3-sialidase sensitive component of each sugar chain from cerebral cortices. Each N-glycan pool was divided into three aliquots and each aliquot treated with neuraminidase or α2,3-sialidase, or buffer as a control experiment, respectively. Each aliquot was then applied to Mono Q HPLC and the flow-through fraction was obtained. N-glycans were separated and quantified using NP- and RP-HPLC. Neuraminidase sensitive component (C^α2,3/6/8^) = N-glycan from a neuraminidase treated aliquot - that from a buffer treated/N-glycan in a neuraminidase treated aliquot × 100. α2,3-sialidase sensitive component (C^α2,3^) was obtained in a similar manner. Values indicate the percentages of the sialidase-sensitive component. The terminal structures of N-glycans are indicated on the extreme left. N.D., not detected

No.	Abbreviation	E12		E16		P0		P7		12W	
		C^α2,3/6/8^	C^α2,3^	C^α2,3/6/8^	C^α2,3^	C^α2,3/6/8^	C^α2,3^	C^α2,3/6/8^	C^α2,3^	C^α2,3/6/8^	C^α2,3^
1	A2G2	N.D.	N.D.	100	4.8	99.5	2.9	91.8	13.1	95.0	6.3
2	A2G2F	95.7	25.6	100	11.0	100	21.9	100	29.4	100	15.3
3	A2G’2F	N.D.	N.D.	91.4	23.4	100	33.6	100	17.1	100	13.8
4	A4G4F	100	44.3	100	38.8	98.6	46.3	100	60.7	100	25.9
5	Ga-BA-2	48.2	<0.1	64.5	18.8	58.0	15.5	54.8	26.6	68.8	28.9
6	Gb-BA-2	34.4	20.5	42.6	25.5	50.3	38.5	45.1	42.5	42.4	35.9
7	G2-BA-2	45.4	<0.1	85.9	3.7	83.5	14.5	68.9	35.5	82.6	34.9
8	A2G1FoF	<0.1	<0.1	<0.1	<0.1	<0.1	<0.1	<0.1	<0.1	<0.1	<0.1
9	A2G2Fo(6)F	76.0	17.6	92.0	39.9	91.1	40.5	90.1	51.7	85.0	54.1
10	A2G2Fo(6)FB	N.D.	N.D.	73.8	18.0	69.3	27.3	70.3	27.4	84.8	18.1
11	A3G3Fo2F	N.D.	N.D.	N.D.	N.D.	N.D.	N.D.	N.D.	N.D.	92.0	15.1
12	Lewis X(a + b)-BA-2	<0.1	<0.1	<0.1	<0.1	<0.1	<0.1	<0.1	<0.1	<0.1	<0.1
13	Lewis X2-BA-2	<0.1	<0.1	<0.1	<0.1	<0.1	<0.1	<0.1	<0.1	<0.1	<0.1
14	Lewis X-H4FB	<0.1	<0.1	<0.1	<0.1	<0.1	<0.1	<0.1	<0.1	<0.1	<0.1
15	Lewis X-H5F	<0.1	<0.1	<0.1	<0.1	<0.1	<0.1	<0.1	<0.1	<0.1	<0.1

### Analysis of the linkages of sialic acid residue on N-glycans during brain development

**Fig. 4:**
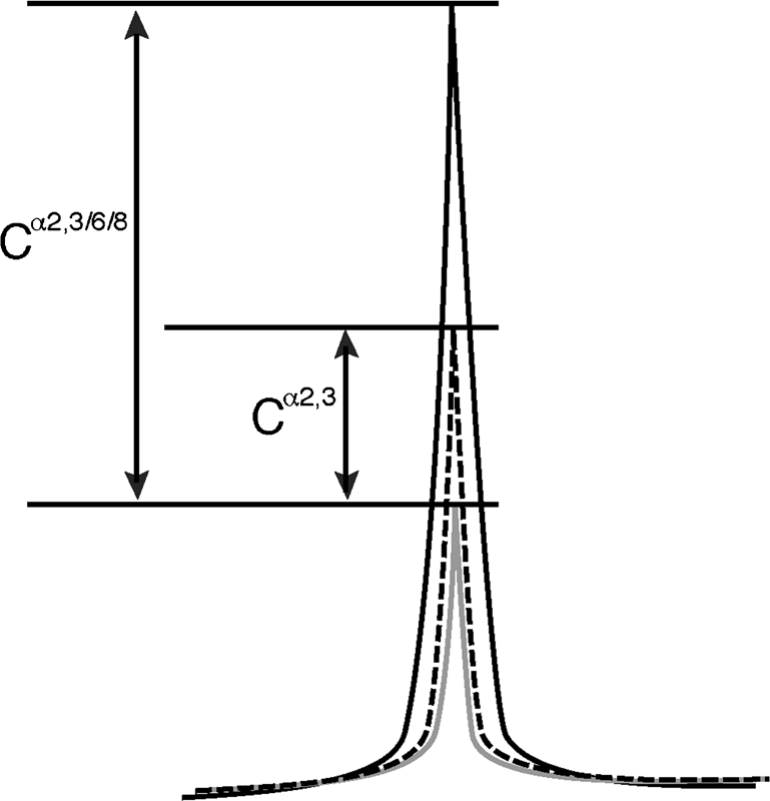
Schematic drawing of the HPLC chart. The *black line* indicates the chromatogram of neutral and desialylated PA-oligosaccharides and the *gray line* indicates that of neutral sugar chains. From the composite images of two chromatograms, sialylated sugar chains can be calculated as the difference in the peak areas (C^**α**2,3/6/8^ value). *Dotted line* shows a chromatogram obtained after α2,3-sialidase treatment of the sample. α2,3-sialidase sensitive portion (C^α2,3^ value) can be obtained from the difference in the peak areas under the *dotted* and *gray lines*

To determine the linkages of sialic acid residues on N-glycans, an analysis was performed using α2,3-sialidase (specific for α2,3-NeuAc). PA-N-glycans were treated with α2,3-sialidase in place of neuraminidase and analyzed as described before, through Mono Q HPLC separation to RP-HPLC. Examples of HPLC profiles used for further calculations are shown in Fig. 4. When all of the sialic acid residues are connected to N-glycans *via* an α2,3-linkage, the neuraminidase sensitive portion (C^α2,3/6/8^ value) and α2,3-sialidase sensitive portion (C^α2,3^ value) should be the same. Quantification of the α2,3-sialidase sensitive proportion of the major sialylated N-glycans is presented in Table 3. From this table the α2,3-sialidase resistant portion can also be calculated by subtracting the C^α2,3^ value from the C^α2,3/6/8^ value. This difference should indicate the amount of N-glycans containing sialic acid with an α2-6/8-linkage. Therefore, it is highly likely that the mouse brain contains large amounts of N-glycans containing sialic acid with an α(2-6)-linkage. However, this conclusion contrasts with a report by Zamze *et al*. [8] that 3-substituted galactose is far more abundant than 6-substituted galactose in the adult rat brain according to a methylation analysis. To determine whether mouse brain contains an abundant amount of N-glycans with a NeuAcα(2-6)-Gal- moiety, SNA lectin affinity chromatography was performed. The initial PA-sugar chain mixture from the P0 mouse brain was applied to the SNA lectin column and the adsorbed fraction was further subjected to Mono Q HPLC analysis. Most of the sugar chains were recovered in the S1 and S2 fractions (data not shown). After neuraminidase treatment, the structures of the sugar chains in these fractions were determined by 2D-HPLC analyses. N-glycans were abundantly present in these fractions, including A2G2, A2G2F, Ga-BA-2, Gb-BA-2 and G2-BA-2. These were all type 2 sugar chains containing a Galβ(1-4)-GlcNAc- structure. Interestingly several N-glycans such as Ga/b-BA-2, G2-BA-2 and A2G2Fo(6)F contained very little or no α2,3-sialidase sensitive portions in the E12 mouse brain, but these increased during development (Table 3), suggesting a switch from α2,6-sialylation to α2,3-sialylation on the same N-glycan.

### Identification of a N-glycan structure containing branched α2,6-sialic acid residue in the mouse brain

It is known that there are two types of α2,6-sialylated N-glycans: one attached to the galactose residue at the non-reducing end (NeuAcα(2-6)-Gal-) and the other to the GlcNAc residue in the type 1 antennary of N-glycans [Galβ(1-3){NeuAcα(2-6)}GlcNAc-]. Since there was only one major type 1 N-glycan (A2G’2F) in the mouse cortex, we determined the linkage type of sialic acid residues on A2G’2F in the adult mouse cortex.

A2G’2F has four potential sialylation sites (two on Gal and two on GlcNAc). After Mono Q HPLC fractionation and neuraminidase treatment, A2G’2F was recovered primarily from the S2 and S3 fractions (data not shown), thus A2G’2F generally contains two or three sialic acid residues. We focused on the S2 fraction for further analysis because the amount of A2G’2F in the S2 fraction was higher than that in the S3 fraction. After α2,3-sialidase treatment of the S2 fraction, A2G’2F was recovered from the S0, S1 and S2 fractions (data not shown), indicating that A2G’2F has two α2,3-sialidase-sensitive sialic acids, one resistant and one sensitive sialic acid residue or two α2,3-sialidase-resistant sialic acids.

**Fig. 5:**
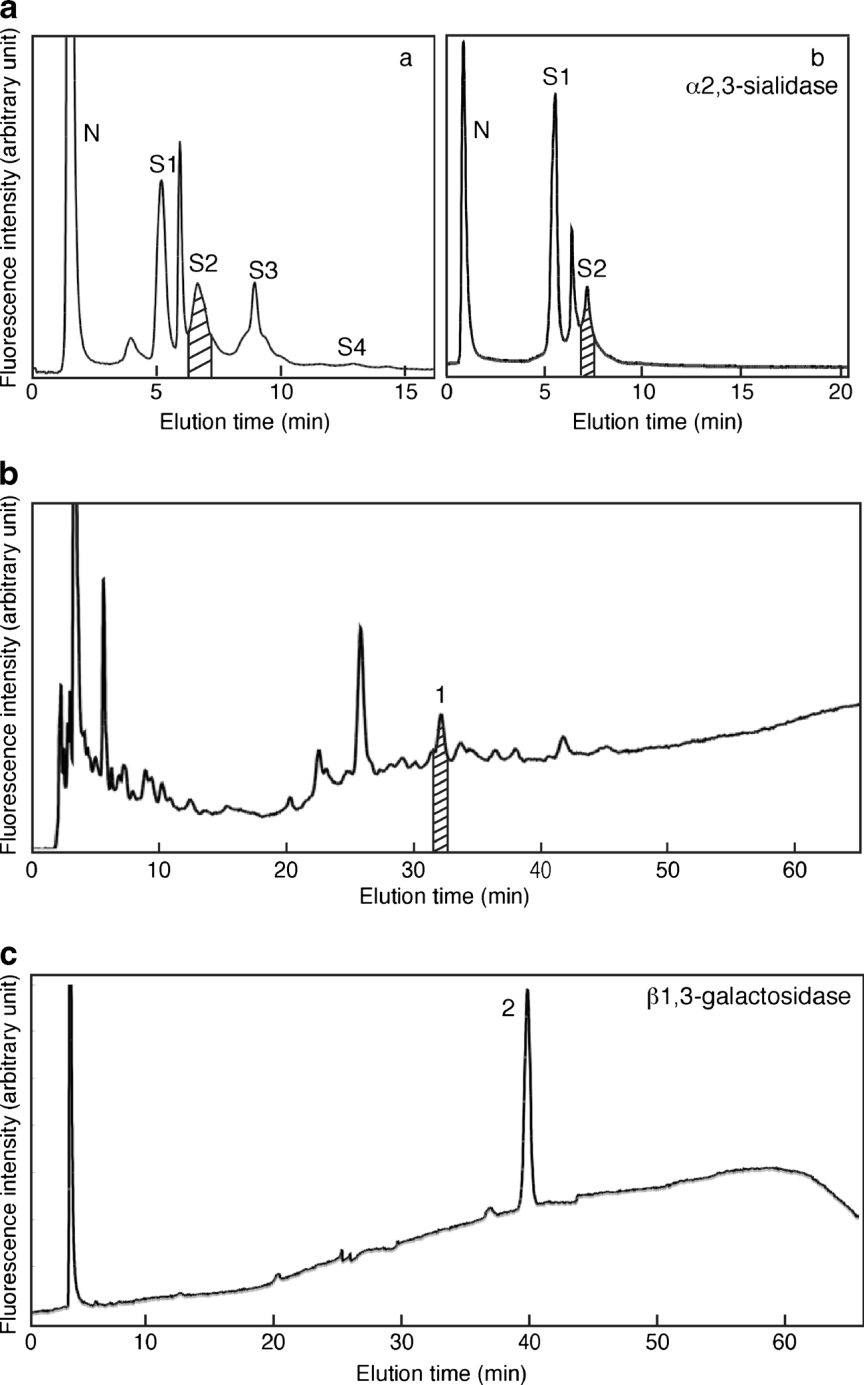
Isolation of di-sialylated A2G’2F **a** separation by Mono Q HPLC of N-glycans from 12w mouse brains. N, S1-S4 indicate the elution positions of neutral, monosialo, disialo, trisialo and tetrasialo PA-N-glycans, respectively. (*a*) N-glycans derived from 12w cerebral cortex were applied again to Mono Q HPLC and the S2 fraction (*indicated by oblique lines*) was collected. (*b*) After sialylated N-glycans from the S2 fraction in Fig. 5a-*a* were treated with α2,3-sialidase, the sample was applied again to Mono Q HPLC. The S2 fraction (*indicated by oblique lines*) was collected **b** the α2,3-sialidase-resistant S2 fraction in Fig. 5a-*b* was applied to an ODS column. There were some major peaks, and the peak 1 was identified as sialylated A2G’2F. The fraction indicated by *oblique lines* was collected. **c** N-glycans from the peak 1 in Fig. 5b were treated with β1,3-galactosidase and applied again to an ODS column. The peak 2 was collected for further analysis. Results are representative of more than three independent experiments

**Fig. 6:**
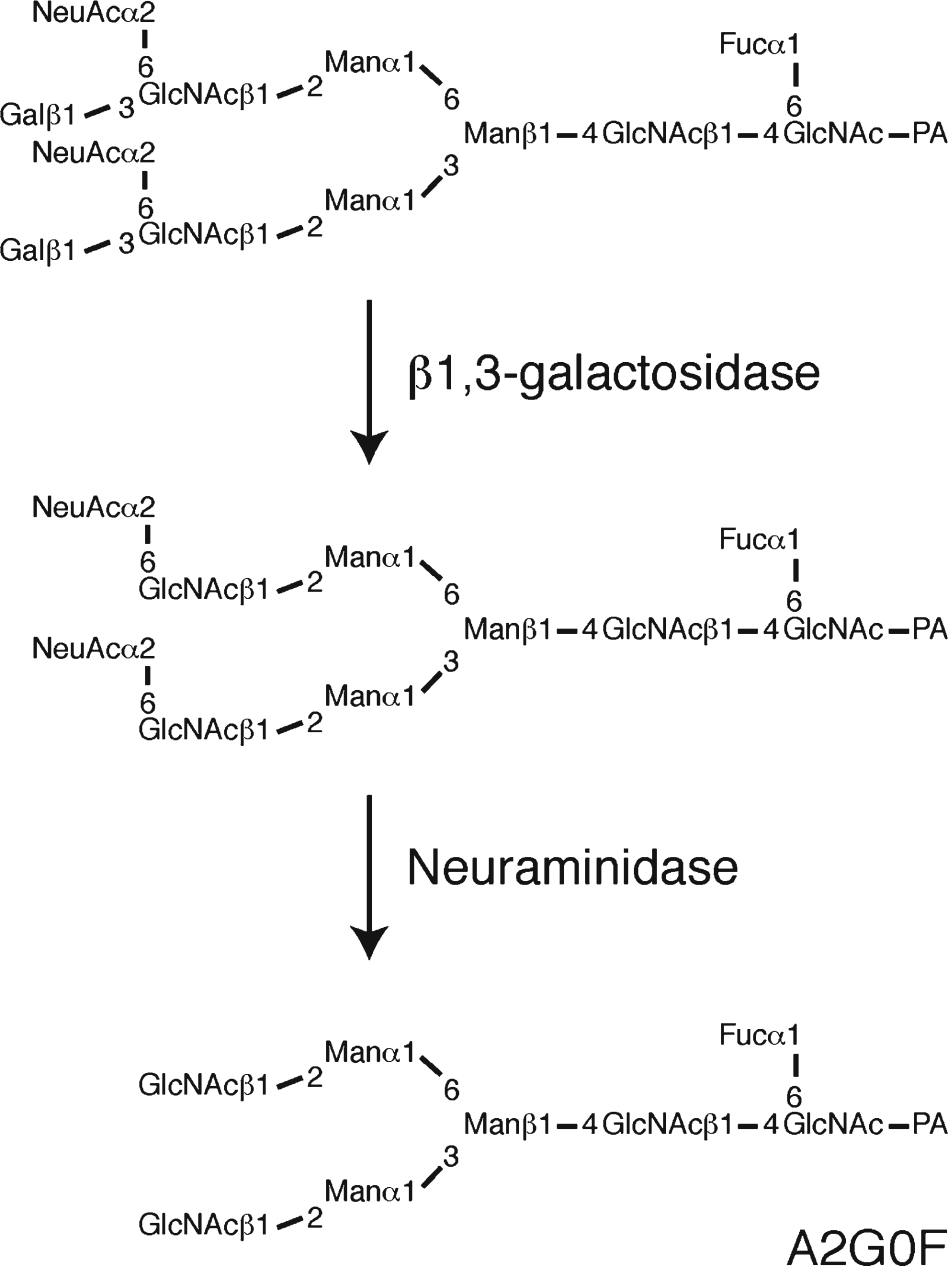
Identification of the N-glycan with 6-sialyl Lewis C. The presumed structure of the N-glycan that was isolated as in Fig. 5 is shown in the upper panel. After β1,3-galactosidase treatment, the galactose-removed sugar chain was collected (*middle panel*), which still harbored two sialic acids as determined by Mono Q HPLC (Supplementary Fig. S1). Finally, this de-galactosylated sugar chain was treated with neuraminidase and its structure determined by 2D-HPLC mapping. The structure turned out to be A2G0F (*lower panel*)

The next step was to determine at which position the α2,3-sialidase-resistant sialic acid residues are attached. We decided to isolate A2G’2F with two α2,3-sialidase-resistant sialic acid residues in its sialylated (native) form. The PA-sugar chain mixture extracted from 12w mouse brains was subjected to Mono Q HPLC and the S2 fraction was recovered (Fig. 5a-*a*). Sugar chains in this fraction were treated with α2,3-sialidase, subjected to Mono Q HPLC and the S2 fraction was collected again (Fig. 5a-*b*). This fraction should contain only sugar chains having two α2,3-sialidase-resistant sialic acid residues. This fraction was separated by RP-HPLC in the presence of triethylamine (Fig. 5b). There were some major peaks in this fraction. Each peak was collected, treated with neuraminidase, and analyzed using NP- and RP-HPLC by comparison with known standards. The peak 1 in Fig. 5b was identified as A2G’2F, thus this sugar chain is the one we desired. The peak 1 was collected and then digested with β1,3-galactosidase to determine if the sialic acid is attached to the terminal galactose residue. After the enzymatic reaction, the elution time for the sugar chain on RP-HPLC with triethylamine changed, indicating that at least one galactose residue had been removed (Fig. 5c). This product (the peak 2) was analyzed by Mono Q HPLC and the results indicated it contained 2 sialic acid residues (Supplementary Fig. S1). After neuraminidase treatment the galactosidase-reaction product was analyzed by 2D-HPLC mapping and was found to be identical to A2G0F (Fig. 6). These results demonstrate that sialic acids were not attached to the terminal galactose residues of the sialylated N-glycan. There is only one possible attachment site for sialic acids other than galactose, *i.e*. the 6th position of the GlcNAc residue. Thus, we propose the structure to be the one presented in Fig. 6. As far as we know, this is the first report identifying α2,6-sialylated GlcNAc-containing N-glycans without terminal galactose sialylation in the brain.

To exclude a possibility of a tandem di-sialyl linkage, α(2-8)-linked di-sialic acid chains, at the 6th position of GlcNAc, the di-sialylated N-glycan from the peak S2 in Supplementary Fig. S1 was treated with β-*N*-acetylhexosaminidase (Supplementary Fig. S2A). After β-*N*-acetylhexosaminidase treatment, the sample was analyzed by Mono Q HPLC and the results indicated it contained 2 sialic acid residues (Supplementary Fig. S2B). The di-sialylated N-glycan from the peak S2 in Supplementary Fig. S2B was treated with neuraminidase. The product was analyzed by 2D-HPLC mapping, NP-HPLC with MU standards (Supplementary Fig. S2C) and RP-HPLC with standards (data not shown), and was found to be identical to A2G0F. These results indicate that di-sialylated A2G’2F contained two [Galβ(1-3){NeuAcα(2-6)}GlcNAc-] structures, not α(2-8)-linked di-sialic acid chains.

**Fig. 7:**
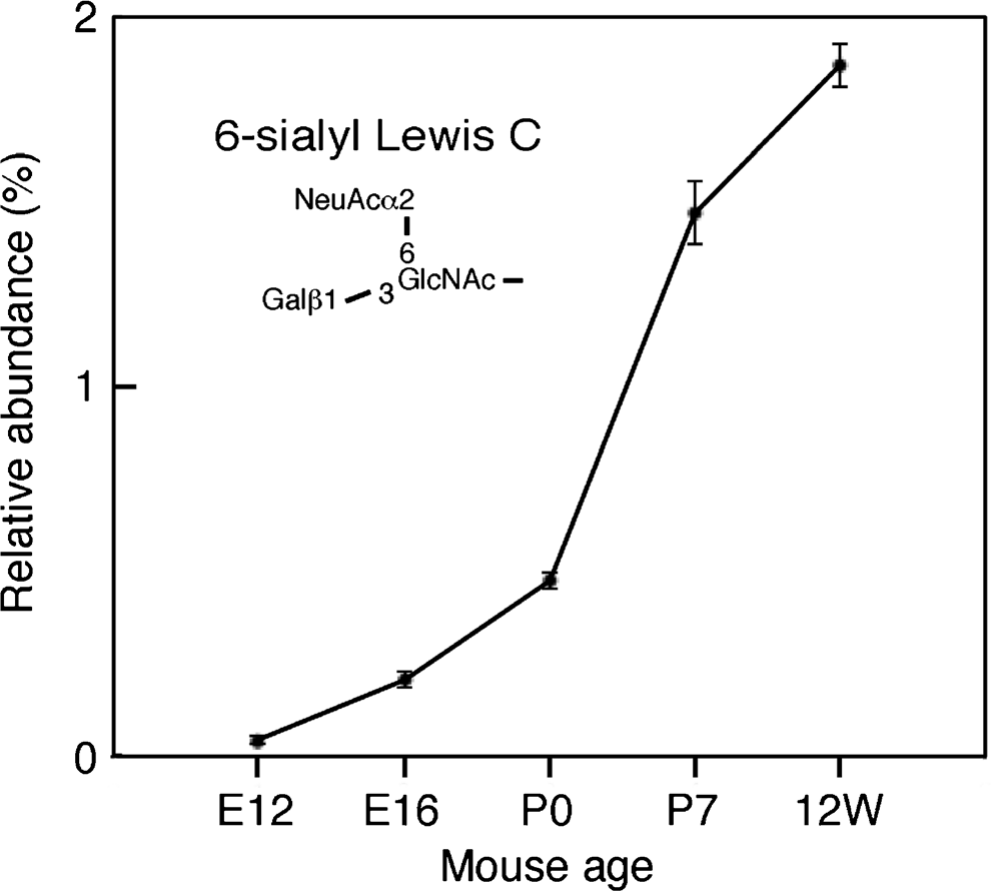
The expression of N-glycans with 6-sialyl Lewis C. The relative abundance of N-linked sugar chains derived from the developing and adult mouse cerebral cortices (E12, E16, P0, P7 and 12w) containing Galβ1,3-(NeuAcα2,6-)GlcNAc- is shown

We examined the expression levels of N-glycans with the branched sialic acid-containing structure [Galβ(1-3){NeuAcα(2-6)}GlcNAc-], termed 6-sialyl Lewis C, in the developing and adult mouse cerebral cortices. Whereas N-glycans with 6-sialyl Lewis C were barely detected at E12, their expression level increased during development, reaching approximately 2 % of the total N-glycan level in the adult as shown in Fig. 7. These results suggest that N-glycans with 6-sialyl Lewis C plays important roles during brain development.

## Discussion

It is essential to determine the entire structure of the sialylated sugar chain, including all of the linkages between sugar residues, to understand roles played by siglecs and sialylated sugar chains. It is also important to know their expression levels during development. A previous study by Zamze *et al*. [8] reported a comprehensive analysis of sialylated N-glycans in the adult rat brain. However, several crucial points were missing: 1) Linkages of sialic acids attached to each N-glycan were not analyzed; 2) Only adult brains, but not developing brains, were analyzed; 3) Quantification of N-glycans was based on MALDI-TOF mass spectrometric analysis, thus expression levels of different sugar chains could not be compared with each other. To overcome these problems, we analyzed developing and adult mouse brains by fluorescently labeling N-glycans followed by HPLC analysis. Neuraminidase and α2,3-sialidase were used to determine the linkages of the sialic acid attachments. Through our analysis the entire structure and expression levels of all N-glycans whose content exceeds 0.1 % of the total N-glycan content were elucidated.

### Expression changes of sialylated N-glycans during mouse brain development

This study uncovered mainly three types of linkage between sialic acid and N-glycans: 1) NeuAcα(2-3)-Galβ(1-4)-GlcNAc-, 2) NeuAcα(2-6)-Galβ(1-4)-GlcNAc-, 3) Galβ(1-3)-{NeuAcα(2-6)}GlcNAc-. Some of the N-glycans were found to change their type of sialic acid linkage during brain development. A good example is Ga-BA-2, which has only one possible sialic acid attachment site. This sugar chain harbors no sialic acid attached *via* an α(2-3)-linkage at E12, but in the adult about half of the N-glycan harbors a NeuAcα(2-3)-Gal- moiety (Table 3). Whereas several N-glycans such as Ga/b-BA-2, G2-BA-2 and A2G2Fo(6)F contained very little or no α2,3-sialidase sensitive portions in the E12 mouse brain, these expression levels were increased during brain development. Since α(2-3)- and α(2-6)-sialic acid moieties are recognized by different siglecs [13, 14, 29], they would be expected to play distinct roles during brain development. Siglecs known to be present in the brain, such as MAG, L1, neurofascin, contactin and many others recognize the NeuAcα(2-3)-Gal- structure (reviewed in Kleene and Schachner [16]), and thus the developmental appearance of NeuAcα(2-3)-Gal- structure should have physiological significance.

We previously reported that the expression level of A2G’2F is increased during brain development [3]. Here, we showed that the expression level of structure 3), 6-sialyl Lewis C, was increased during development in the mouse brain (Fig. 7). A2G’2F with 6-sialyl Lewis C is a branched structure but in the rat brain the same backbone (A2G’2F) harbors the di-sialyl Lewis C [NeuAcα(2-3)-Galβ(1-3)-{NeuAcα(2-6)}GlcNAc-] epitope and usually bears 4 sialic acid residues [8]. We initially suspected that the sialic acid residue attached to the galactose residue within the Lewis C epitope on A2G’2F had been artificially removed during our N-glycan preparation procedure. Therefore, we started from a standard PA-sugar chain containing the di-sialyl Lewis C epitope (Takara Bio, PA-sugar chain 025) and followed the same preparation procedure from hydrazinolysis to pyridylamination. The di-sialyl Lewis C epitope was recovered in a totally intact form (data not shown), thus this branched structure is not an artifact. We examined tri-sialyl N-glycans in Fig. 5a-*a*, and found that A2G’2F with both 6-sialyl Lewis C and di-sialyl Lewis C (manuscript in preparation). To determine whether there is a great difference in the structures of sialyl Lewis C containing N-glycans between rat and mouse cortices at the type 1 antennary, N-glycan structures in rat cortices were analyzed using the same method applied to analyze mouse cortices. Cortices from both species were found to contain 6-sialyl Lewis C and di-sialyl Lewis C at similar levels (manuscript in preparation). Therefore, we concluded that 6-sialyl Lewis C is also present in the rat brain, where it should play an important role during brain development.

### Potential roles of 6-sialyl Lewis C

It was reported that some glycoproteins in bovine plasma possess N-glycans with 6-sialyl Lewis C [30–32]. However, physiological roles of 6-sialyl Lewis C and siglecs that recognize this motif have not been known yet. A mono-sialyl ganglioside containing 6-sialyl Lewis C, Galβ(1-3){NeuAcα(2-6)}GlcNAcβ(1-3)Galβ(1-4)Glcβ1,1-ceramide; LS-tetrasaccharide b (LSTb)-ceramide, was reported [33, 34]. LSTb-probes bind to Siglec-7 better than Siglec-9 [35]. Even though the significance of these in the brain is unknown because LSTb has only been described in human milk [33, 34], it is possible that 6-sialyl Lewis C plays important roles with specific siglecs in the brain. It remains to be determined whether or not siglecs that bind to the di-sialyl Lewis C epitope or the 6-sialyl Lewis C epitope are distinct from each other. It would be interesting to examine roles of 6-sialyl Lewis C by using synthesized and clustered 6-sialyl Lewis C probes [36]. Further studies are necessary to address these issues.

## Electronic supplementary material

Below is the link to the electronic supplementary material.

ESM 1 (PDF 605 kb)

## Abbreviations

Fuc: fucose
Gal: galactose
GlcNAc: *N*-acetylglucosamine
GU: glucose unit value on the reverse-phase column
HPLC: High performance liquid chromatography
MALDI-TOF-MS: matrix assisted laser desorption/ionization time-of-flight mass spectrometry
Man: mannose
MU: mannose unit value on the normal-phase column
NeuAc: *N*-acetylneuraminic acid
ODS: octadecylsilyl

## References

[1] Lau KS, Partridge EA, Grigorian A, Silvescu CI, Reinhold VN, Demetriou M, Dennis JW (2007). Complex N-glycan number and degree of branching cooperate to regulate cell proliferation and differentiation. Cell.

[2] Ohtsubo K, Marth JD (2006). Glycosylation in cellular mechanisms of health and disease. Cell.

[3] Ishii A, Ikeda T, Hitoshi S, Fujimoto I, Torii T, Sakuma K, Nakakita S, Hase S, Ikenaka K (2007). Developmental changes in the expression of glycogens and the content of N-glycans in the mouse cerebral cortex. Glycobiology.

[4] Ngamukote S, Yanagisawa M, Ariga T, Ando S, Yu RK (2007). Developmental changes of glycosphingolipids and expression of glycogenes in mouse brains. J. Neurochem..

[5] Hakomori S (1981). Glycosphingolipids in cellular interactions, differentiation and oncogenesis. Annu. Rev. Biochem..

[6] Miyoshi E, Nishikawa A, Ihara Y, Gu J, Sugiyama T, Hayashi N, Fusamoto H, Kamada T, Taniguchi N (1993). N-acetylglucosaminyltransferase III and V messenger RNA levels in LEC rats during hepatocarcinogenesis. Cancer Res..

[7] Varki A (2010). Uniquely human evolution of sialic acid genetics and biology. Proc. Natl. Acad. Sci. U. S. A..

[8] Zamze S, Harvey DJ, Chen YJ, Guile GR, Dwek RA, Wing DR (1998). Sialylated N-glycans in adult rat brain tissue. A widespread distribution of disyalylated antennae in complex and hybrid structures. Eur. J. Biochem..

[9] Mühlenhoff M, Rollenhagen M, Werneburg S, Gerardy-Schahn R, Hildebrandt H (2013). Polysialic acid: versatile modification of NCAM, SynCAM 1 and neuropilin-2. Neurochem. Res..

[10] Dallérac G, Rampon C, Doyère V (2013). NCAM function in the adult brain: lessons from mimetic peptides and therapeutic potential. Neurochem. Res..

[11] Bakhti M, Snaidero N, Schneider D, Aggarwal S, Möbius W, Janshoff A, Eckhardt N, Nave KA, Simons M (2013). Loss of electrostatic cell-surface repulsion mediates myelin membrane adhesion and compaction in the central nervous system. Proc. Natl. Acad. Sci. U. S. A..

[12] Eggers K, Werneburg S, Schertzinger A, Abeln M, Schiff M, Scharenberg MA, Burkhardt H, Mühlenhoff M, Hildebrandt H (2011). Polysialic acid controls NCAM signals at cell-cell contacts to regulate focal adhesion independent from FGF receptor activity. J. Cell Sci..

[13] Crocker PR (2002). Siglecs: sialic-acid-binding immunoglobulin-like lectins in cell-cell interactions and signaling. Curr. Opin. Struct. Biol..

[14] Fischer E, Brossmer R (1995). Sialic acid-binding lectins: submolecular specificity and interaction with sialoglycoproteins and tumour cells. Glycoconj. J..

[15] Attrill H, Takazawa H, Witt S, Kelm S, Isecke R, Brossmer R, Ando T, Ishida H, Kiso M, Crocker PR, van Aalten DM (2006). The structure of siglec-7 in complex with sialosides: leads for rational structure-based inhibitor design. Biochem. J..

[16] Kleene R, Schachner M (2004). Glycans and neural cell interactions. Nat. Rev. Neurosci..

[17] Schachner M, Bartsch U (2000). Multiple functions of the myelin-associated glycoprotein MAG (siglec-4a) in formation and maintenance of myelin. Glia.

[18] Vyas AA, Blixt O, Paulson JC, Schnaar RL (2005). Potent glycan inhibitors of myelin-associated glycoprotein enhance axon outgrowth *in vitro*. J. Biol. Chem..

[19] Schengrund CL (1990). The role(s) of gangliosides in neural differentiation and repair: a perspective. Brain Res. Bull..

[20] Yu RK, Nakatani Y, Yanagisawa M (2009). The role of glycosphingolipid metabolism in the developing brain. J. Lipid Res..

[21] Krusius T, Finne J (1977). Structural features of tissue glycoproteins. Fractionation and methylation analysis of glycopeptides derived from rat brain, kidney and liver. Eur. J. Biochem..

[22] Hase S, Ikenaka K, Mikoshiba K, Ikenaka T (1988). Analysis of tissue glycoprotein sugar chains by two-dimensional high-performance liquid chromatographic mapping. J. Chromatogr..

[23] Fujimoto I, Menon KK, Otake Y, Tanaka F, Wada H, Takahashi H, Tsuji S, Natsuka S, Nakakita S, Hase S, Ikenaka K (1999). Systematic analysis of N-linked sugar chains from whole tissue employing partial automation. Anal. Biochem..

[24] Tanabe K, Ikenaka K (2006). In-column removal of hydrazine and N-acetylation of oligosaccharides released by hydrazionolysis. Anal. Biochem..

[25] Yoshimura T, Yamada G, Narumi M, Koike T, Ishii A, Sela I, Mitrani-Rosenbaum S, Ikenaka K (2012). Detection of N-glycans on small amounts of glycoproteins in tissue samples and sodium dodecyl sulfate-polyacrylamide gels. Anal. Biochem..

[26] Otake Y, Fujimoto I, Tanaka F, Nakagawa T, Ikeda T, Menon KK, Hase S, Wada H, Ikenaka K (2001). Isolation and characterization of an N-linked oligosaccharide that is significantly increased in sera from patients with non-small cell lung cancer. J. Biochem..

[27] Hase S (1994). High-performance liquid chromatography of pyridylaminated saccharides. Methods Enzymol..

[28] Audry M, Jeanneau C, Imberty A, Harduin-Lepers A, Delannoy P, Breton C (2011). Current trends in the structure-activity relationships of sialyltransferases. Glycobiology.

[29] Crocker PR, Paulson JC, Varki A (2007). Siglecs and their roles in the immune system. Nat. Rev. Immunol..

[30] Mizuochi T, Yamashita K, Fujikawa K, Kisiel W, Kobata A (1979). The carbohydrate of bovine prothrombin. Occurrence of Gal beta 1 leads to 3GlcNAc grouping in asparagine-linked sugar chains. J. Biol. Chem..

[31] Mizuochi T, Yamashita K, Fujikawa K, Titani K, Kobata A (1980). The structures of the carbohydrate moieties of bovine blood coagulation factor X. J. Biol. Chem..

[32] Mizuochi T, Taniguchi T, Fujikawa K, Titani K, Kobata A (1983). The structures of the carbohydrate moieties of bovine blood coagulation factor IX (Christmas factor). J. Biol. Chem..

[33] Smith DF, Zopf DA, Ginsburg V (1978). Fractionation of sialyl oligosaccharides of human milk by ion-exchange chromatography. Anal. Biochem..

[34] Prieto PA, Smith DF (1985). A new ganglioside in human meconium detected by antiserum against the human milk sialyloligosaccharide, LS-tetrasaccharide b. Arch. Biochem. Biophys..

[35] Yamaji T, Teranishi T, Alphey MS, Crocker PR, Hashimoto Y (2002). A small region of the natural killer cell receptor, Siglec-7, is responsible for its preferred binding to α2,8-disialyl and branched α2,6-sialyl residues. A comparison with Siglec-9. J. Biol. Chem..

[36] Bao GM, Tanaka K, Ikenaka K, Fukase K (2010). Probe design and synthesis of Galβ(1–>3) [NeuAcα(2–>6)]GlcNAcβ(1–>2)Man motif of N-glycan. Bioorg. Med. Chem..

