# Supplementary material for: Determination of major sialylated N-glycans and identification of branched sialylated N-glycans that dynamically change their content during development in the mouse cerebral cortex
Source: Glycoconj J. 2014 Nov 23;31(9):671–83. doi: 10.1007/s10719-014-9566-2 (PMC4245497; doi:10.1007/s10719-014-9566-2)

Supplemental Information

Determination of major sialylated N-glycans and identification of branched sialylated N-glycans that dynamically change their content during development in the mouse cerebral cortex

Tomohiro Torii[1,2,a†], Takeshi Yoshimura[1,2†], Mai Narumi[1,2], Seiji Hitoshi[1,2,b], Yoshie Takaki[3], Shuichi Tsuji[3], and Kazuhiro Ikenaka[1,2]

[1]Department of Physiological Sciences, School of Life Sciences, The Graduate University for Advanced Studies (SOKENDAI), Shonan Village, Hayama, Kanagawa 240-0193, Japan

[2]Division of Neurobiology and Bioinformatics, National Institute for Physiological Sciences, National Institutes of Natural Sciences, 5-1 Higashiyama, Myodaiji-cho, Okazaki, Aichi 444-8787, Japan

[3]Institute of Glycoscience, Tokai University, 4-1-1 Kitakaname, Hiratsuka, Kanagawa 259-1292, Japan

[a]Present address: Department of Pharmacology, National Research Institute for Child Health and

Development, 2-10-1 Okura, Setagaya, Tokyo 157-8535, Japan

[b]Present address: Department of Integrative Physiology, Shiga University of Medical Science, Seta Tsukinowa-cho, Otsu 520-2192, Japan

[†]These authors contributed equally to this work.

Address correspondence to: Kazuhiro Ikenaka, Ph.D.

Division of Neurobiology and Bioinformatics, National Institute for Physiological Sciences, National Institutes of Natural Sciences, 5-1 Higashiyama, Myodaiji-cho, Okazaki, Aichi 444-8787, Japan

Tel.:+81-564-59-5245; Fax: +81-564-59-5247; e-mail: ikenaka@nips.ac.jp

Supplementary Figure legends

Supplementary Fig. S1 Mono Q HPLC chromatogram of N-glycan from the peak 2. N-glycan from the peak 2 in Fig. 5C was applied to an Mono Q column. There was one major peak in the S2 fraction.

Supplementary Fig. S2 Confirmation of di-sialylated A2G’2F. (A) To exclude the possibility that di-sialylated A2G’2F contains a tandem di-sialyl linkage at the 6th position of GlcNAc, the sample was treated with β-N-acetylhexosaminidase after β1,3-galactosidase digestion. After neuraminidase treatment, the structure turned out to be A2G0F because the sialic acid at the 6th position of the GlcNAc residue prevented β-N-acetylhexosaminidase digestion. (B) Separation by Mono Q HPLC of N-glycans after β-N-acetylhexosaminidase treatment. The S2 fraction was collected. (C) N-glycans from the peak S2 in Supplementary Fig. S2B were analyzed by NP-HPLC (a) with MU standards (b). The peak 3 was collected for further analysis.

Experimental Procedures

β-N-acetylhexosaminidase digestion

PA-N-glycans were treated with β-N-acetylhexosaminidase (recombinant gene from *Streptococcus pneumoniae*, expressed in *E. coli*, Prozyme) for 30 min at 37°C according to the manufacturer's instructions. These samples were heated at 100°C for 5 min. The reaction mixture was centrifuged at 2,300 x g for 10 min, followed by filtering through a 0.20 μm spin filter (Ultrafree-MC LG, Millipore).

N-glycan analysis by NP-HPLC

PA-N-glycans were separated by HPLC using a NP-column (Shodex Asahipak NH2P-50 4B, 4.6 x 50 mm, Showa Denko K.K.) at a flow rate of 0.6 ml/min at 30 ºC. The mobile phase consisted of solvent A (93% acetonitrile, 0.3% acetic acid titrated to pH 7.0 with 1 M aqueous ammonia) and solvent B (20% acetonitrile, 0.3% acetic acid titrated to pH 7.0 with 1 M aqueous ammonia). PA-N-glycans were detected at an excitation wavelength of 310 nm and an emission wavelength of 380 nm using a fluorescence detector.

Supplementary Fig. S1

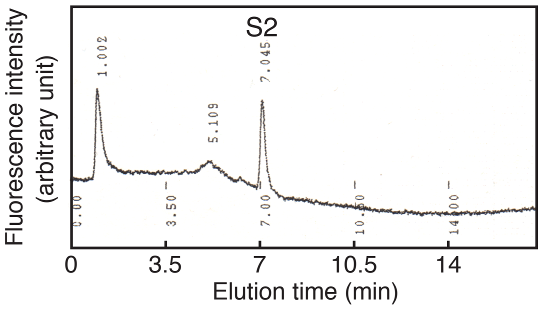

Supplementary Fig. S2

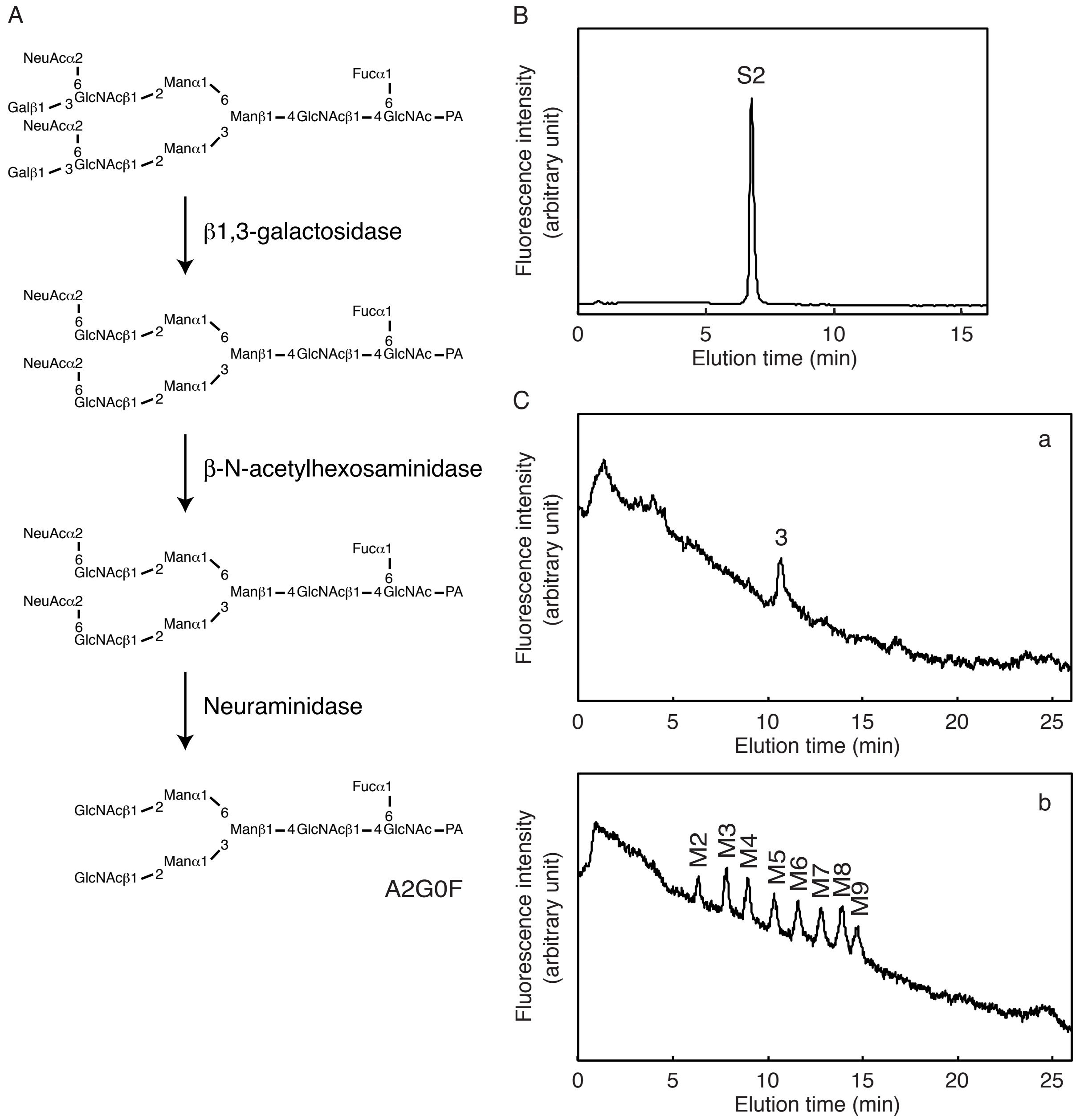

Supplement: Supplementary file 1 — (PDF 605 kb) [file 10719_2014_9566_MOESM1_ESM.pdf]
